# Effects of Elbow Crutch Locomotion on Gluteus Medius Activation During Stair Ascending

**DOI:** 10.3389/fbioe.2022.890004

**Published:** 2022-05-25

**Authors:** Carlos De la Fuente, Alejandro Neira, Gustavo Torres, Rony Silvestre, Matias Roby, Roberto Yañez, Sofia Herrera, Virgina Martabit, Isabel McKay, Felipe P. Carpes

**Affiliations:** ^1^ Departamento de Cs. de la Salud, Facultad de Medicina, Pontificia Universidad Católica de Chile, Santiago, Chile; ^2^ Laboratory of Neuromechanics, Universidade Federal do Pampa, Uruguaiana, Brazil; ^3^ Servicio de Biomecánica, Centro de Innovación, Clínica MEDS, Santiago, Chile; ^4^ Escuela Kinesiología, Facultad de Ciencias, Universidad Mayor, Santiago, Chile; ^5^ Traumatología, Clínica MEDS, Santiago, Chile

**Keywords:** Gluteus Medius, EMG, assistive device, stairs, locomotion

## Abstract

Crutches can help with the locomotion of people with walking disorders or functional limitations. However, little is known about hip muscle activation during stair ascending using different crutch locomotion patterns in people without disorders and limitations. Thus, we determined the acute effects of elbow crutch locomotion on gluteus medius (GM) activity during stair ascending. This comparative analytic cross-sectional study enrolled ten healthy men (22.0 ± 0.47 years). Participants climbed up the stairs with elbow crutches using one or two crutches, with ipsilateral or contralateral use, and after loading or unloading a limb. EMG signals were recorded from anterior, middle, and posterior portions of the GM and compared between the crutch conditions. The Kruskal–Wallis test and Dunn’s multiple comparison test were performed (*α* = 5%). The activation of the GM increased with the ipsilateral use of crutches, with two crutches and three points, and when all the load depended only on one limb. GM activation decreased with contralateral use and in the unload limb. In conclusion, ascending stairs with elbow crutches alters the GM activation. The more critical factors were choosing the crutches’ lateral use, the number of crutches, and if the limb is loaded or unloaded while ascending the stairs. Our findings can be helpful to increase or decrease the GM activation for those who use or will use crutches.

## Introduction

The elbow or Canadian crutch is a nonaxillary assisted device often used in rehabilitation (Rasouli and Reed, 2020). Its design combines forearm and axillary support, providing stability through the upper extremity with the elbow flexed (Rasouli and Reed, 2020). Locomotion with crutches tries to assist users with daily life tasks and movement of patients during rehabilitation, disability, aging, and recovery from acute injuries (Sanders et al., 2018). For instance, the use of crutches by the elderly can help with their locomotion and increase their independence (Harper et al., 2018). Nevertheless, there are substantial effects on energy expenditure and mechanical work assumptions when elbow crutches are used during locomotion (Thys et al., 1996; Halsey et al., 2012).

Although crutches can assist with the locomotion of people with walking disorders or limitations, the use of crutches may affect activation muscle patterns, i.e., muscles acting for frontal plane stabilization. In this regard, a recent study found a negative relation between gluteus medius (GM) activation and dynamical stability (margins-of-stability) using poles during walking (Peyré-Tartaruga et al., 2022). The change in the activation of muscles acting in the frontal plane has critical functional implications for the hip and pelvis stability (Semciw et al., 2016; Buckthorpe et al., 2019). The GM stabilizes the pelvis and controls the hip adduction and internal rotation (Ebert et al., 2017). Unfortunately, crutch locomotion may alter the GM activity during weight-bearing and unipedal postures (Jebsen, 1967; Hsue and Su, 2010; Ebert et al., 2017), leading to altered lower limb joint control. The most used locomotion pattern is one crutch on the contralateral side to increase the support base, providing better balance and reducing mediolateral displacement of the center of mass (Jebsen, 1967; Hsue and Su, 2010). However, there are many locomotion patterns with crutches combining the delay between the crutches and foot placement (when the foot lands after the crutch), the number of concurrent points of contact (total number of points in contact with the ground), laterality (one crutch moving at a time), or the load/unload of the limb (Rasouli and Reed, 2020).

For example, walking stairs requires additional strength, a range of motion, balance, and coordination compared with gait at the ground level (Hsue and Su, 2010). However, little is known about how the neuromuscular system responds to the increased demand. Stair ascent involves a hip extension combined with adduction followed by abduction and forward contralateral rotation of the pelvis while the center of mass ascents against gravity (Hsue and Su, 2010; Hall et al., 2017). Elbow crutches increase the mediolateral displacement of the center of mass when walking up the stairs (Hsue and Su, 2010) since it might influence GM activity. Previous studies have addressed the effects of crutch use on energy expenditure, ground reaction forces during locomotion at the ground level, and the activity of ankle and knee muscles (Carpentier et al., 2001; Halsey et al., 2012; Moran et al., 2015; Watanabe et al., 2018; Rasouli and Reed, 2020). However, little is known about the GM activity during stair ascent. When not using crutches, the GM is activated later at the instant of the foot landing on the stairs (Brindle et al., 2003). Its function is pelvis and hip stabilization to reduce the center of mass oscillation (Hsue and Su, 2010; Benedetti et al., 2012).

The knowledge about GM activation while walking up the stairs using different crutch locomotion patterns is important to help manage activation exercises and acquire occupational skills for those experiencing long- and short-term use of crutches (Ebert et al., 2017) since understanding how GM can acutely adapt its neuromuscular activity is a fundamental first step. Therefore, here, we aimed to determine the effect of elbow crutch locomotion on GM muscle activity during stair ascending. We hypothesized that GM activation depends on choosing the crutches’ lateral use, the number of crutches, and if the limb is loaded or unloaded while ascending the stairs.

## Materials and Methods

### Study Design

To study the effects of elbow crutch locomotion on GM muscle activity during stair ascending, we have focused our study on nonimpaired people who can provide us with typical neuromuscular adaptations of the nervous and the motor system. Participants were recruited from the local community. Each participant provided written consent to be included in this study, which was approved by ethics committee no. 200721006 from the Pontificia Universidad Católica de Chile and conducted following the Declaration of Helsinki.

### Participants

The volunteers should be men aged between 18 and 25 years old, achieving at least 72 points in the lower extremity functional scale (Repo et al., 2017) and having a fully functional joint range of motion in the sagittal plane. Data were collected from 10 healthy men with an age of 22.0 ± 0.47 years, body mass of 68.8 ± 10.9 kg, height of 1.70 ± 0.06 m, BMI of 23.8 ± 3.3 kg/m^2^, and lower extremity functional scale score of 79.0 ± 1.5 points, and participants without any orthopedic commitment or postural alteration including cavus and flat foot and without experience with the use of elbow crutches in the past participated in this cross-sectional study. As a similar experimental setup and population were not found in the literature, we measured the post hoc power. We measured the post hoc power in multiples of 5, and we stopped the recruitment when the post hoc power was equal to or more than 0.80.

Participants were oriented to come to the laboratory using comfortable clothes (to avoid limitation in the range of motion), to avoid wearing shoes with a height higher than 4 cm (Hapsari and Xiong, 2016), to not fast for more than 4 h, to refrain from vigorous physical exercise, to not take alcohol in the 72 h preceding the tests, and to sleep for at least 6 h in the night before the tests. Participants were excluded if they reported pain or discomfort of any type during the trials. For data collection, they performed different patterns of elbow crutch locomotion while ascending stairs at a self-selected speed. The neuromuscular activation from the GM was recorded while performing stair ascent with different patterns of locomotion with crutches.

### Staircase Design and Crutch

**FIGURE 1:**
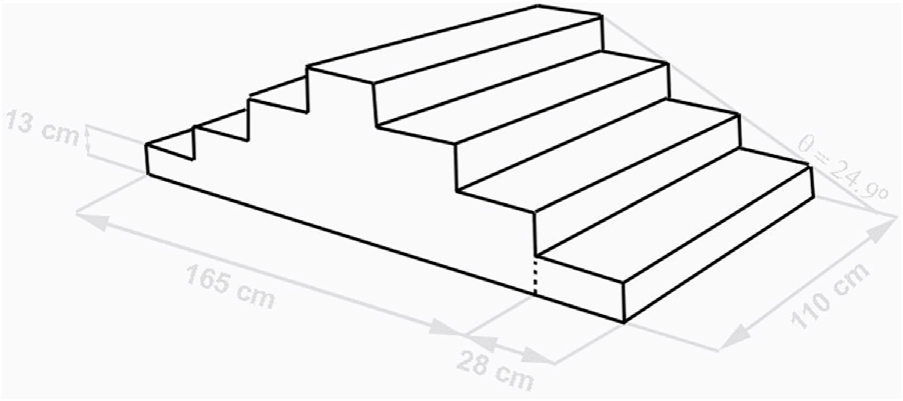
Staircase dimensions. The experimental environment conditions of the study.

The motor task consisted of ascending a wood custom-made staircase with four symmetrical steps built according to the standards described in National Law of Housing and Urbanism no. 1305 (Chilean Mistry, 1992), imitating the local conditions of the stairs. The step had a rise of 13 cm, run of 28 cm, and a width of 110 cm, resulting in a stair angle (slope) of 24.9° (Figure 1). The participants used an aluminum elbow crutch with a rubber tip and a plastic forearm cuff and handle (Figure 1). The height was defined as the vertical distance from the ground to the greater trochanter anatomical reference, with 20° of elbow flexion (Hsue and Su, 2010).

### Crutch Locomotion

Two weeks before data collection, all participants were familiarized with the measurements and the locomotion using crutches as well as control conditions. The familiarization sessions were performed three times a week, lasted 1 h, and were always conducted by the same researcher. The familiarization was performed to ensure correct learning of the locomotion pattern of stair ascent with and without (control condition) the elbow crutches. During each familiarization session, locomotion was videotaped to show the patterns used and to reinforce the learning. Before data collection, all participants achieved the criteria of fluid locomotion, no falls, and complete foot contact on the steps (Inoue et al., 2012).

**FIGURE 2:**
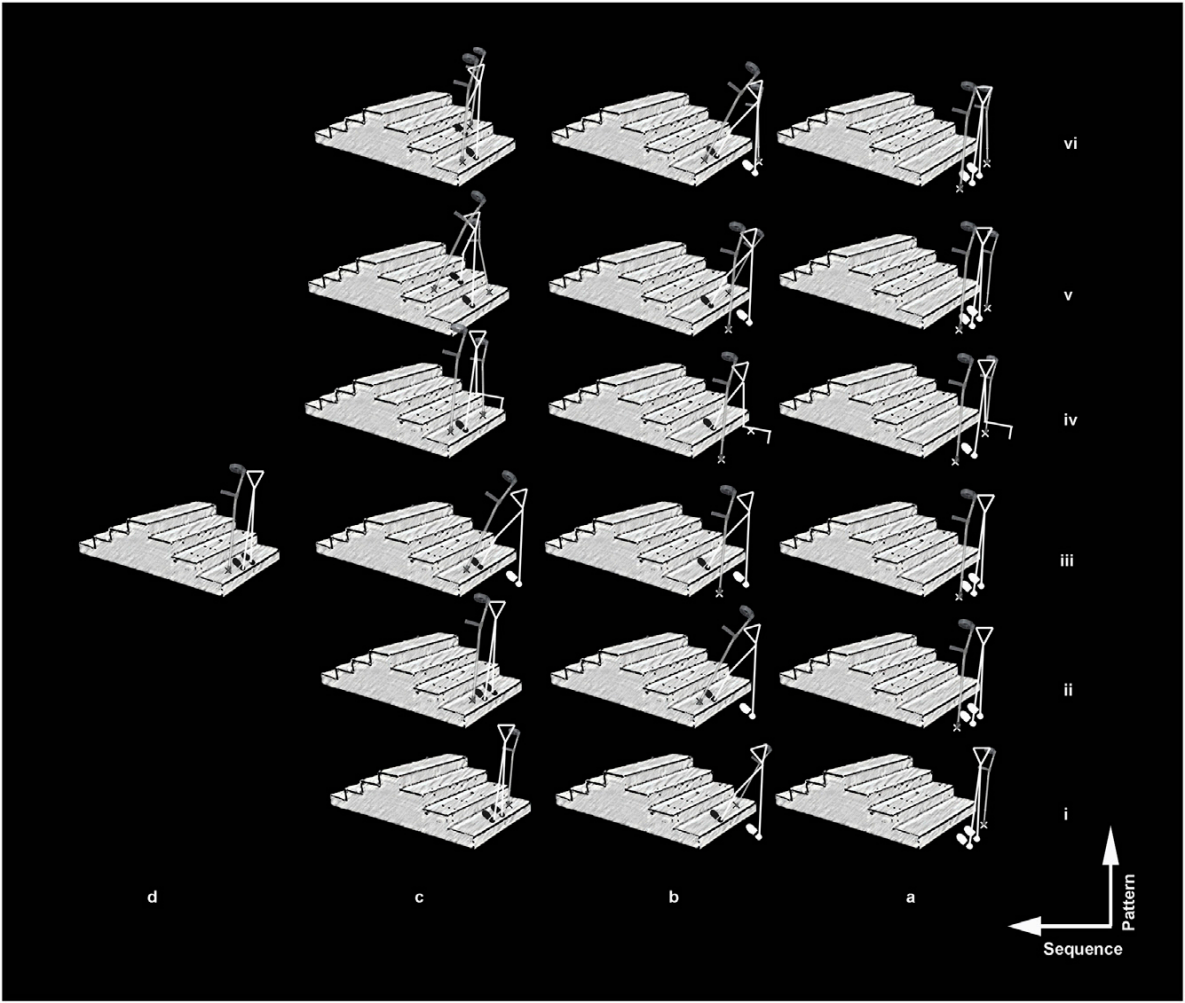
Crutch locomotion patterns. The image shows a half of the kinematic patterns using crutches in the study (*n* = 6) from i to vi. The total number of crutch locomotion patterns was 12, which resulted from changing the limb side to start the movement. In addition, the figure shows the movement sequence from letter a to d. Finally, the weight-bearing of the limb and crutch over the ground is indicated with a print stick figure and Canadian crutches. The stair ascending control (without crutches, *n* = 2) were omitted.

For data collection, the participant was bipedally positioned, standing in the front and 28 cm away from the first step of the stairs. Following a verbal “go” command, the participant ascended the stairs at a self-selected speed (Hsue and Su, 2010). In this study, twelve experimental conditions and two control conditions that resulted in fourteen conditions in total were studied (see Figure 2 and Table 1), with 10 min of rest in between. The ascending was performed in a random order to reduce fatigue effects (Hsue and Su, 2010). All conditions were tested on the same day to avoid the effects of EMG sensor shift. Three valid trials, which mean no loss of control and balance during ascending, were considered for data analysis. In addition, a control ascending was added, obtaining two control conditions, one for each limb.

**TABLE 1:** Details of the different conditions for crutch locomotion during stair ascending.

Crutch condition	Support Base	Crutch side	Movement and conditions
One-crutch	Three-points	Right	Left foot stepped up first with a contralateral crutch (condition 1) followed by the right foot (condition 8)
One-crutch	Three-points	Left	Left foot stepped up first with an ipsilateral crutch (condition 9) followed by the right foot (condition 2)
One-crutch	Three-points	Left	Left foot stepped up first without a crutch followed by a delayed ipsilateral crutch (condition 3), after which the right foot is stepped up (condition 10)
Two-crutches	Three-points	Both sides	Left foot stepped up first without crutches followed by two-crutches (condition 4), while the right leg is unloaded (condition 11)
Two-crutches	Four-points	Both sides	Left foot stepped up first with a contralateral crutch (condition 5) followed by the right foot with a contralateral crutch (condition 12)
Two-crutches	Four-points	Both sides	Left foot stepped up first with an ipsilateral crutch (condition 6) followed by the right foot with a contralateral crutch (condition 13)
Control	Two-points	Without	The locomotion control strategy was added, establishing two conditions for limbs and normal stair walking: left foot stepped up first (condition 7) followed by the right foot (condition 14)

### Measurements

Data with respect to age, height, BMI, and lower extremity functional scale (Repo et al., 2017) were collected during the familiarization sessions. The neuromuscular activity was recorded by surface electromyography (EMG) from the anterior, middle, and posterior portions of the GM. With the participant standing, the surface electrodes for the anterior region were placed at 50% of the distance between the anterosuperior iliac spine and the greater trochanter for the anterior fiber of the GM, the electrodes for the middle fibers were placed at 50% of the distance between the greater trochanter and the iliac crest, and the electrodes for the posterior fibers were placed at 33% of the distance between the posterior ilium and the greater trochanter (Otten et al., 2015). Electrodes were placed after the skin was shaved and cleaned according to the “Surface Electromyography for the Non-Invasive Assessment of Muscles” guidelines (Hermens et al., 2000). After the placement of each electrode, the participant was requested to perform a maximal isometric test to check the selective action of the GM according to the sensor placement (Semciw et al., 2014; Otten et al., 2015). The quality of the signal was checked visually before any recording.

### EMG Acquisition and Treatment

The raw EMG signals were collected using a Trigno Wireless 16-Channel EMG system (Delsys Inc., Boston, United States) and with CMRR >80 dB, a gain of 1,000, an interelectrode distance of 10 mm, and a sampling rate of 2000 Hz through the software Nexus 1.8.5 (Vicon Motion Systems Ltd., United Kingdom). For offline processing, the EMG signals were mean-centered and filtered with a band-pass of 20–450 Hz with a second-order Butterworth filter (Semciw et al., 2014). The magnitude of muscle activation was estimated by applying a root mean square envelope with a window length of 250 ms and sliding 1 sample using a custom-made script written in Matlab 2016a software (Mathwork Inc., United States). The ICA algorithm was used to separate the independent basal noise of the whole EMG signals (Zheng and Hu, 2019). The EMG signals were normalized to each lower limb to the respective peak root mean square (rms) value obtained during the control condition for stair ascent (Dwyer et al., 2013).

### Statistical Analysis

Data normality was checked using the Shapiro–Wilk test. EMG data showed a nonparametric distribution. Therefore, these are reported as median ± interquartile range. The muscle activation between the different gluteus medius regions was compared using the one-way Kruskal–Wallis test and Dunn’s multiple comparison test with an alpha of 5% after Bonferroni’s correction due to normality assumptions. The *p*-values were summarized using a connection graph to better understand the multiple statistical differences between conditions (Bastian et al., 2009). All statistical analyses were performed using the Matlab software 2016a (Mathworks, Inc., United States). The posteriori statistical power was estimated using G*Power software version 3.1.9.2 (Universitat Dusseldorf, Dusseldorf, Germany).

## Results

EMG data were normalized to the control condition from each limb. The anterior GM activation was higher under conditions 1, 2, 3, 4, 5, 6, 9, and 13 than under control conditions. The middle portion of the GM showed higher activation under conditions 1, 3, 4, 6, 9, and 13 than under control conditions. The posterior portion of the GM showed higher activation under conditions 2, 3, 4, 6, 9, and 13 than under control conditions. In contrast, the other conditions for all regions showed lower median values than the control conditions.

**FIGURE 3:**
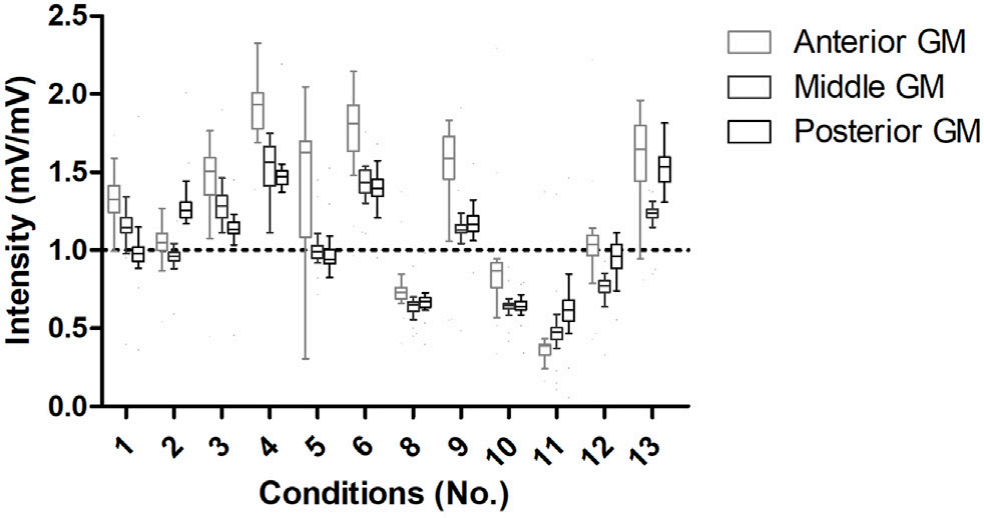
EMG intensity of crutch patterns locomotion. The horizontal line indicates the median reference of the control condition. When EMG intensities are above and below the horizontal line, there was more and less activation for the portions of the GM than under the control condition, respectively. GM, gluteus medius.

**FIGURE 4:**
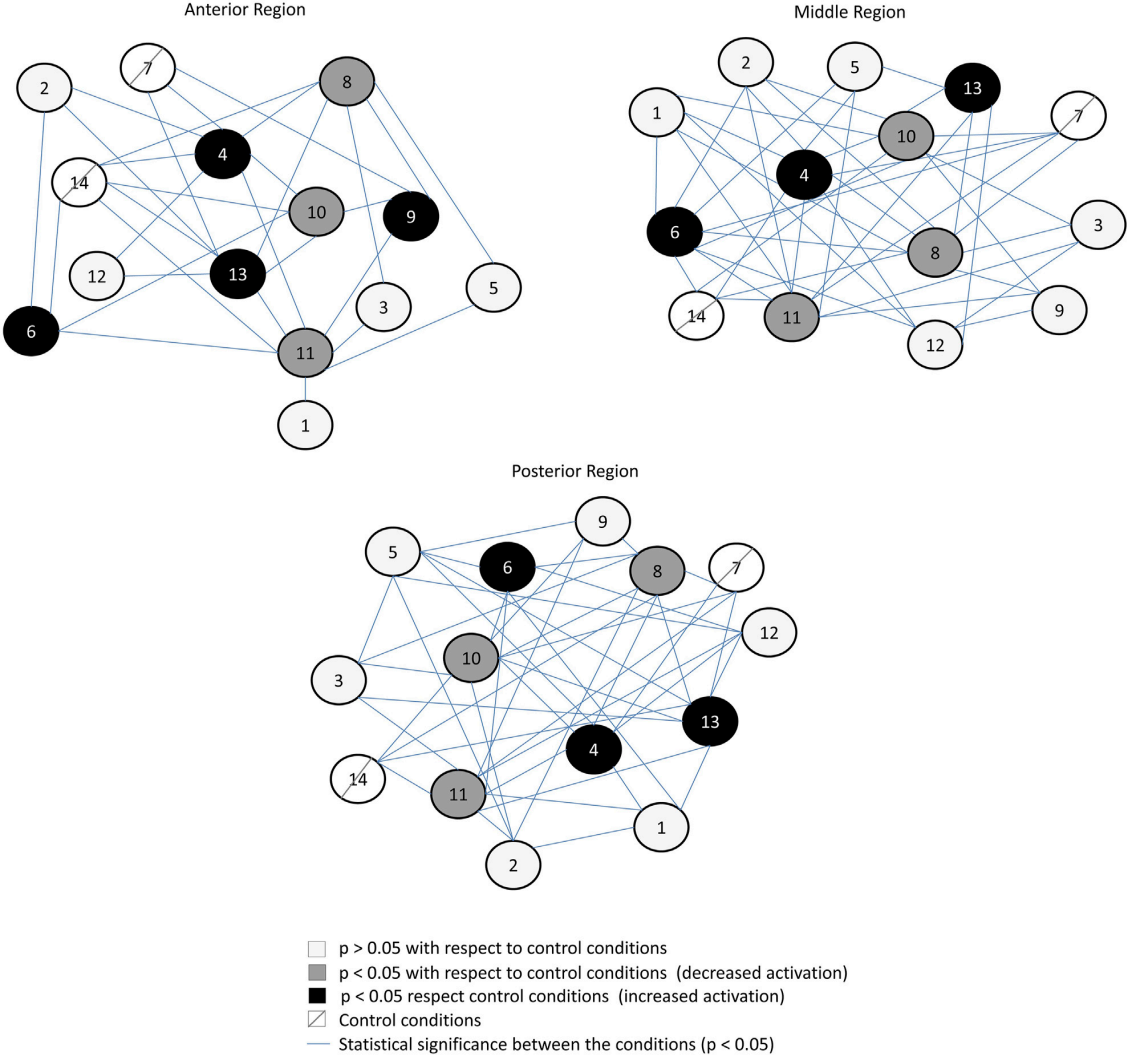
Connection graph of the statistical differences found between conditions and regions of the gluteus medius. Conditions 7 and 14 are the control conditions (ascending stairs starting with the left and right limb, respectively).

Activation of anterior (*p* < 0.001), middle (*p* < 0.001), and posterior (*p* < 0.001) portions of the GM increased with the ipsilateral use of crutches and in the loaded extremity using two crutches with three support points (see Figures 3, 4). In contrast, activation of anterior (*p* < 0.001), middle (*p* < 0.001), and posterior (*p* < 0.001) portions of the GM decreased in the unloaded extremity and also when one-contralateral crutch supports the ascent of the stairs or provides support in a contralateral manner while the other limb ascends (see Figures 3, 4). The posteriori statistical power was 0.99.

## Discussion

In this study, we demonstrate that different locomotion crutch patterns affect the GM muscle activity during stair ascending because changes in rms with respect to the control conditions were observed. The rms of the EMG signal is a measure of muscle excitation caused by the sarcolemma depolarization of activated motor units, which is the main component of the EMG signal and the result of the sum of different muscle fiber excitations (Vigotsky et al., 2017). Thus, our findings can be grouped by the lateral use of crutches (higher activation for ipsilateral use), the number of crutches (higher activation with two crutches and three points), and the load/unload of the extremity (higher activation in the loaded limb). The contralateral use and unloaded limb reduced GM activation. As far as we know, this is the first study to determine the effects of the different patterns of locomotion using elbow crutches during stair ascent on GM activation. Our findings have important implications for guiding the management of the crutch locomotion for the community.

Higher GM activation was found for stair ascent using two crutches with three points in the loaded limb (Figure 2, see 4th condition). This pattern is frequently recommended in clinics for “going up with the uninvolved limb first” (Jebsen, 1967). The increase in GM activation is in coherence with the increase in mechanical work required for body displacement with a unilateral stance when the center of mass is located further back, such as that observed for squat exercises that also increase GM activity (Bolgla et al., 2016; Ebert et al., 2017).

The stair ascent using two ipsilateral crutches with four points also increased GM activity. This pattern implies two symmetrical conditions (6th and 13th conditions) where the ipsilateral crutch acts as a pivot, is closer to the center of mass, and permits the contralateral limb to oscillate while it ascents to the next step (Jebsen, 1967; Hsue and Su, 2010). Studies simulating gait found that hip abductor weakness results in compensatory movements of the pelvis (Borrelli and Haslach, 2014). We hypothesize that higher GM activation likely occurred to provide pelvis stability in the frontal plane while rotating forward during the ascent movement (Jebsen, 1967; Heim et al., 2000; Lee et al., 2014) and more significant mediolateral displacement of the center of mass (Hsue and Su, 2010), similar to the strategies used during unilateral stance tasks (Bolgla et al., 2016; Ebert et al., 2017; Moore et al., 2019).

Mechanical constraints due to the use of crutches also account for higher GM activation. Under the ipsilateral crutch condition, the perpendicular distance between the crutches and the GM is lower since it requires a larger hip abductor movement to ascent the stairs (Borrelli and Haslach, 2014). Pelvis hiking has been associated with larger mediolateral displacement of the center of mass and indicates abductor weakness (Svehlík et al., 2012). We consider that identifying the crutch locomotion patterns leading to increased GM activity can help manage muscle activation, for example, in geriatric populations or patients with neuromuscular impairment (Hsue and Su, 2010).

Stair ascent using two crutches with three points resulted in lower GM activity in the unloaded limb. We hypothesize that the GM stabilizing role is combined with the activation of abductors’ muscles to generate the coactivation necessary to avoid excessive movement of the contralateral pelvis associated with larger displacement of the center of mass and instability (Lewis et al., 2017). The other two patterns eliciting lower GM activation occurred in the delayed limb (Figure 2, see 8th and 10th conditions). Both patterns created a triangular base of support using three points with one crutch. The first condition had a higher angle located in the upper step, and the second condition had a higher angle located in the back step during the ascent. Both strategies suggest that the passive moment produced by the crutch reduces GM activity in the delayed limb. Lower muscle activity under this condition agrees with decreased active mechanical work during gait with assistive devices (Ajemian et al., 2004; Borrelli and Haslach, 2014) and the increased base of support provided by the crutch aids (van der Veen et al., 2020). These strategies are taught mainly in physiotherapy schools as options to manage acute lower limb injuries and avoid overload in tissues with low resistance in healing processes resulting from fractures (Sanders et al., 2018).

The higher activity of the anterior region of the GM in most patterns suggests that its recruitment helps control the contralateral forward pelvis rotation during stair ascending (Semciw et al., 2014). The higher activation of this region may result in the challenge to control the forward contralateral pelvis movement during the ascent, whereas the activation of the medial fibers is more related to frontal stabilization (Semciw et al., 2013; Semciw et al., 2014), and posterior fibers stabilize the head of the femur especially because its fiber orientation permits active control of anterior instabilities (Semciw et al., 2013; Safran, 2019). In general, the crutch locomotion patterns elicit more instability, increasing the GM activity, which we interpret as a highlight of the role of this muscle in helping to achieve a more stable movement pattern. Therefore, appropriate conduct would be needed when impaired activation and instability behaviors exist, for example, in patients operated with hip arthroscopies (Safran, 2019) or acute muscle inhibitions due to joint edema (Rice et al., 2014).

Currently, there is a lack of scientific knowledge for the prescription of assistive devices. Our results may help better manage early muscle deficits due to rest under acute injuries (Maguire et al., 2016) or long-time adaptation in the elderly population using assistive devices (Allen, 2001; Allen et al., 2001; Hamel and Cavanagh, 2004). We acknowledge that EMG crosstalk can limit our experiment (Semciw et al., 2014). Another limitation is the movement of the fibers under the skin during hip movements. We used interelectrode distances and the location of sensors standardized according to the literature to minimize these effects. We also conducted a normalization and low level of step rise to control the excessive displacement of the fiber under the skin.

## Conclusion

The more important factors affecting the activation of the motor unit pool of the GM during elbow crutch locomotion for ascending stairs were the lateral use of crutches, the number of crutches, and the load/unload of extremity. Ascending stairs using two crutches with three points in the unloaded limb elicited lower activation than that observed for the loaded extremity. This novel knowledge may have utility in increasing the activity of frontal pelvis–hip stabilizers when crutches are used.

## Data Availability

The datasets presented in this study can be found in online repositories. The names of the repository/repositories and accession number(s) can be found below: https://www.researchgate.net/publication/357092654_Effects_of_different_elbow_crutches_locomotion_patterns_on_anterior_middle_and_posterior_Gluteus_Medius_activation_during_stair_ascending_data.
